# Mind-body therapies intervention for essential hypertension: network meta-analysis based on the antihypertensive effect

**DOI:** 10.3389/fcvm.2026.1677353

**Published:** 2026-02-25

**Authors:** Haojun Cui, Wanru Guo, Hongfei Zhao, Jun Chen

**Affiliations:** 1 Shanghai University of Sport of Martial Arts Academy, Shanghai, China; 2 Shanghai Jiao Tong University, Shanghahi, China; 3 Handan University, Handan, China

**Keywords:** diastolic blood pressure, essential hypertension, mind-body therapies intervention, network meta-analysis, randomized controlled trial, systolic blood ressure

## Abstract

**Aim:**

Hypertension is a major risk factor for cardiovascular and cerebrovascular diseases and may lead to serious health outcomes such as stroke, myocardial infarction, heart failure, and renal failure. Essential hypertension (EH) accounts for approximately 90% of all hypertension cases. In this study, we applied a network meta-analysis (NMA) to quantitatively synthesize evidence from randomized controlled trials (RCTs) and to compare and rank the effects of mind–body therapies (MBTs) on systolic and diastolic blood pressure in patients with EH, with the aim of providing evidence to support informed selection of MBT interventions.

**Methods:**

A comprehensive search strategy restricted to English-language RCTs was developed and applied across multiple biomedical databases, including PubMed, Embase, the Cochrane Library, Web of Science, CBM, China National Knowledge Infrastructure (CNKI), Wanfang, and VIP, from database inception to March 2, 2025. (The literature found in the Chinese database did not meet the requirements.) Two authors independently screened studies, extracted data, and performed a frequentist network meta-analysis using STATA version 18.0 to compare and rank MBTs with respect to their effects on systolic blood pressure (SBP) and diastolic blood pressure (DBP). Methodological quality of the included studies was assessed using the Cochrane Risk of Bias tool. Additional analyses were conducted to evaluate network consistency and robustness of the findings.

**Results:**

A total of 15 RCTs involving 949 participants were included, comprising 18 intervention arms and 11 distinct MBT interventions. While most MBTs demonstrated some degree of blood pressure–lowering effect, SUCRA values indicated that Bhramari Pranayama (BP; SUCRA = 75.3%) and Specific Qigong (SQ; SUCRA = 73.7%) had the highest probabilities of ranking among the more effective interventions for reducing SBP. For DBP reduction, Sound Relax Meditation (SRM; SUCRA = 83.0%), Specific Qigong (SQ; SUCRA = 80.2%), and Bhramari Pranayama (BP; SUCRA = 78.1%) showed relatively favorable rankings. These rankings represent relative probabilities and should be interpreted in conjunction with effect sizes and the overall certainty of evidence.

**Conclusion:**

Compared with other MBTs, Bhramari Pranayama and Specific Qigong demonstrated more favorable relative effects on systolic blood pressure in patients with essential hypertension. Sound Relax Meditation, Specific Qigong, and Bhramari Pranayama showed comparatively better performance in reducing diastolic blood pressure. Given the limited number of studies and modest sample sizes, these findings should be interpreted cautiously, and further large-scale, high-quality RCTs are needed to confirm these comparative effects.

## 1 Introduction

Hypertension is a clinical syndrome characterized by elevated systemic arterial pressure and increased peripheral vascular resistance (1). Despite substantial advances in understanding the pathophysiology of cardiovascular disease (CVD) and the availability of effective therapeutic strategies, essential hypertension remains a major modifiable risk factor for CVD. Elevated blood pressure substantially increases the risk of cardiovascular morbidity and mortality in millions of individuals worldwide, and available evidence suggests that this burden will continue to rise (2). Hypertension affects more than one billion people globally, and this number is projected to increase to 1.5 billion by 2025. The majority of hypertension cases are idiopathic, commonly referred to as essential hypertension (EH). In 2023, the World Health Organization explicitly identified EH as a prevalent chronic condition among the aging population (3). EH is defined by elevated blood pressure of unknown etiology and is associated with an increased risk of cerebrovascular, cardiac, and renal events. In industrialized countries, the lifetime risk of developing hypertension (blood pressure >140/90 mmHg) exceeds 90%. Moreover, EH frequently coexists with other cardiovascular risk factors, including aging, overweight or obesity, insulin resistance, diabetes mellitus, and hyperlipidemia (4).

Current clinical guidelines recommend angiotensin-converting enzyme inhibitors, diuretics, and calcium channel blockers as first-line antihypertensive therapies. Although all antihypertensive agents effectively lower blood pressure—and blood pressure reduction itself is the most important determinant of cardiovascular risk reduction—differences exist among drug classes in their ability to prevent target-organ damage and major cardiovascular events. Most patients with hypertension require combination therapy with two or more antihypertensive agents, often in conjunction with statins, to adequately control blood pressure and associated risk factors. Nevertheless, despite the availability of effective and generally safe pharmacological treatments, blood pressure control remains suboptimal in a large proportion of patients. Long-term pharmacotherapy is also associated with adverse effects such as dizziness, fatigue, orthostatic hypotension, bradycardia, hypokalemia, and poor treatment adherence (5). These limitations underscore the need to explore complementary therapeutic approaches that are widely accessible, cost-effective, and sustainable.

Mind–body therapies (MBTs) are broadly defined as a group of interventions that emphasize the integration of mental and physical processes to facilitate healing (6). MBTs are grounded in the concept that interactions among the brain, mind, body, and behavior play a critical role in health and disease, and that emotional, psychological, social, and spiritual factors can exert direct physiological effects (7). Over recent decades, MBTs—including tai chi, qigong, meditation, and yoga—have attracted increasing scientific attention, with growing interest in evaluating their safety and effectiveness (8). Many of these interventions are rooted in ancient traditions and are widely used to alleviate symptoms and improve overall health. Furthermore, MBTs may be implemented either as standalone interventions or as adjuncts to conventional medical treatment, as enhanced self-efficacy itself may confer physiological benefits (9).

In this systematic review, we employed a network meta-analysis (NMA) approach, which enables the simultaneous comparison of multiple interventions within a single analytical framework by integrating direct and indirect evidence from randomized controlled trials (RCTs). In contrast, traditional pairwise meta-analyses are limited to synthesizing studies designed for direct comparisons between two interventions.

Network meta-analysis is a versatile methodology that allows for the concurrent comparison of multiple interventions (e.g., A vs. B, B vs. C) and facilitates estimation of their relative effects within an evidence network (10). By combining both direct and indirect comparisons, NMA provides a comprehensive assessment of comparative effectiveness and supports informed decision-making regarding optimal intervention selection.

Accordingly, the present study aimed to evaluate the comparative effects of multiple MBTs on essential hypertension using NMA. Specifically, we analyzed RCTs investigating yoga (including compositive yoga breathing, Nadi Shodhana Pranayama, Bhramari Pranayama, Sheetali Pranayama, and Trataka), meditation (sound relax meditation and non-sound relax meditation), qigong (specific qigong and compositive qigong), and tai chi as interventions for EH.

Although numerous systematic reviews and meta-analyses have previously examined the effectiveness of various non-pharmacological or physical interventions for EH, most focused on only two or a limited number of treatments. Many prior reviews included non-randomized studies, lacked quantitative synthesis, or primarily compared MBTs with placebo, wait-list controls, or routine care. Consequently, the available evidence has been insufficiently comprehensive and lacks precise comparative outcome metrics to delineate the relative effects of different MBTs on blood pressure reduction. As a result, existing literature provides limited guidance regarding the comparative effectiveness of MBTs in lowering SBP and DBP, despite substantial variability among interventions in both cost and therapeutic impact.

To address these gaps, the present study systematically reviewed available RCTs, reclassified MBTs into more granular and clinically meaningful subtypes, and compared and ranked their effects on SBP and DBP in patients with essential hypertension. By providing a more detailed and integrative comparative assessment, this NMA aims to offer additional evidence to support clinicians and patients in selecting appropriate MBT interventions as part of hypertension management.

## 2 Methods

### 2.1 Eligibility criteria and literature search

We searched the following databases for RCTs from the beginning to March 2, 2025: PubMed, Embase, Cochrane, Web of Science, CINAHL, CBM, China National Knowledge Infrastructure (CNKI), Wanfang, and VIP in compliance with the Preferred Reporting Items for Systematic Reviews and Meta-analyses (PRISMA) statement and the Cochrane Collaboration extension statement (11). Only studies published in English were eligible for inclusion. Chinese databases (CNKI, Wanfang, and VIP) were searched to identify English-language publications indexed within these platforms. At the same time, because there is no available English literature in the Chinese database, the database has been revised and cut off. Search strings included a combination of Medical Subject Headings (MeSH terms or Emtree terms) and free-text terms related to EH (“Essential Hypertension”, “Hypertension, Essential”, “Primary Hypertension”, “Hypertension, Primary”, “Hypertension”, “Blood Pressure”), MBTs (“Mind-body Therapies”, “Mind-Body Medicine”, “Mind-Body MBTs”, “Meditation”, “Transcendental Meditation”, “Mindfulness”, “Tai Ji”, “Tai Chi”, “Tai Chi Chuan”, “Qigong”, “Ch'i Kung”, “Yijinjing”, “Wuqinxi”, “Liuzijue”, “Baduanjin”, “Yoga”) and RCTs (“randomized controlled trial”, “random control”, “placebo”). The MeSH terms and free words were linked by “OR” within each group, and the groups were linked by “AND” for the search.

### 2.2 Study selection criteria

The inclusion criteria for this study was strictly adhered to the PICOS framework, and the inclusion criteria are as follows. (1) Study design was published RCTs available in English as the primary language for the treatment of EH; (2) Participants: participants that were diagnosed with EH with SBP ≥ 140 mmHg and/or DBP ≥ 90 mmHg; (3) Interventions: the treatment group received MBTs; (4) Comparison: the control group received routine care; (5) Outcomes: changes in SBP and DBP after treatment (blood pressure change = prior treatment blood pressure value−posttreatment blood pressure value).The primary outcomes were systolic blood pressure (SBP) and diastolic blood pressure (DBP) measured at the end of the intervention period. Post-intervention mean values and standard deviations were used for the primary network meta-analysis, as change-from-baseline data were inconsistently reported across included trials. When both baseline and post-intervention values were available, change scores were extracted for exploratory sensitivity analyses. However, due to incomplete reporting in several studies, change-from-baseline outcomes could not be used uniformly and were therefore not selected as the primary outcome metric. This approach assumes approximate baseline comparability between randomized groups and is consistent with prior network meta-analyses when change scores are unavailable.

The exclusion criteria are as follows: (1) Nonrandomized controlled study; (2) Participants with secondary hypertension or serious complications, including “congestive heart failure (NYHA Class III/IV), end-stage renal disease (eGFR <30 mL/min/1.73 m^2^), stroke within 6 months, myocardial infarction, or malignant hypertension (SBP ≥180 mmHg and/or DBP ≥120 mmHg with acute organ damage)”; (3) Duplicated publications; (4) participants or intervention not corresponding to our inclusion criteria; (5) Data cannot be extracted; (6) Studies that failed to reported the baseline which results in an inability to calculate changes in SBP and DBP after treatment; (7) Studies that only reported antihypertensive efficiency rate but failed to provide specific numerical values; (8) Full text was not available; (9) Meeting abstracts, protocols, animal experiments, and reviews.

**Figure 1:**
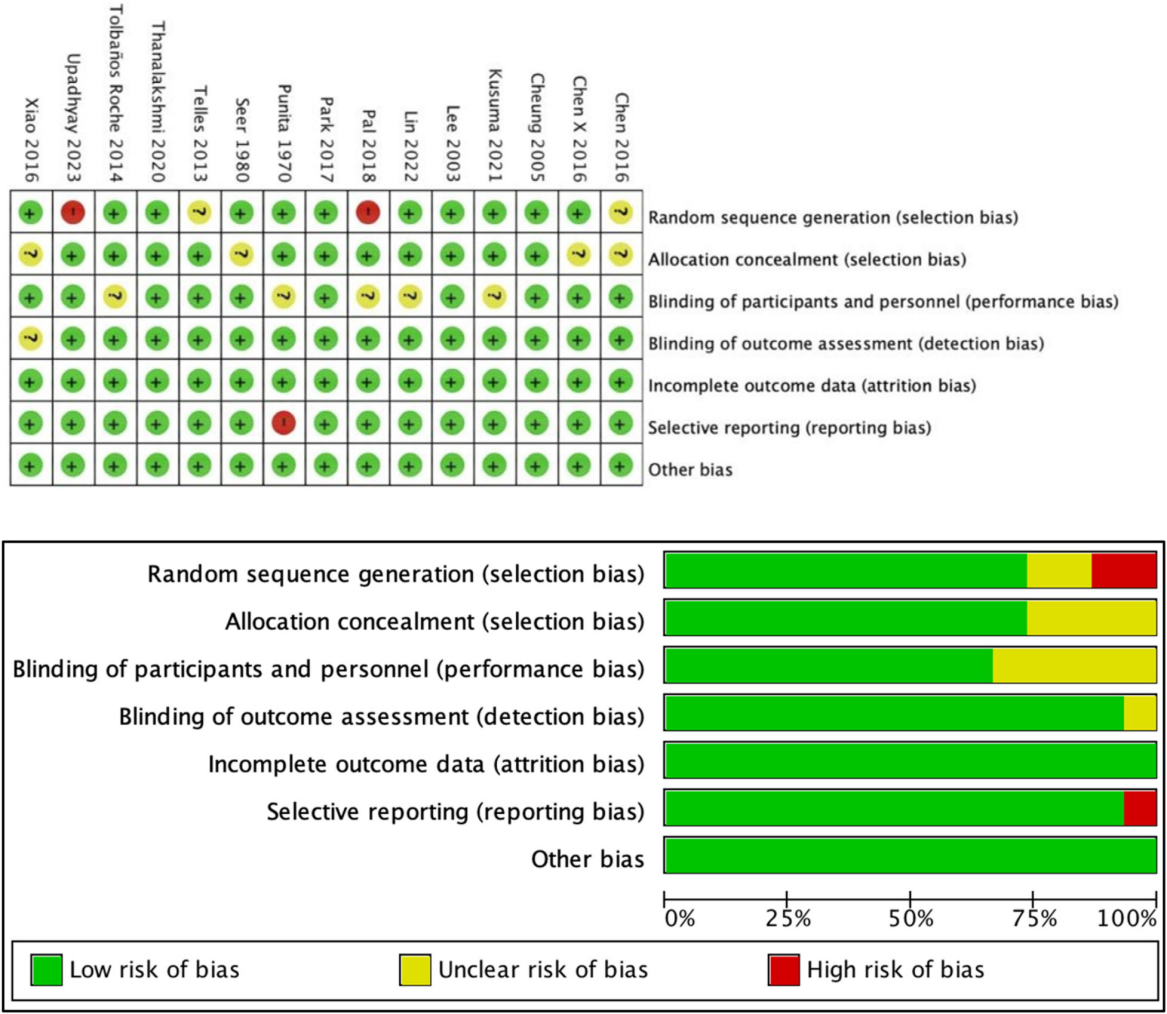
Analysis of the risk of bias in accordance with the Cochrane collaboration guideline.

Titles, abstracts, and texts were independently evaluated for possible inclusion by two authors (GWR and CHJ), who then debated their differences until they were agreed upon. The first author (GWR) retrieved the data, and among the extracted parameters were the kind, frequency, and duration of the MBTs intervention, as well as participant characteristics (sample size, age, disease duration, and SBP or DBP scores at baseline and post-intervention). Finally, the reference lists of included articles were searched for eligible studies. Six potential sources of bias (risk of bias, RoB) were assessed for each RCT using the revised Cochrane Collaboration Tool (12): (1) bias from the randomization process, (2) deviation from intended interventions, (3) missing outcome data, (4) bias from the outcome measurement, (5) selective outcome reporting, and (6) overall bias. The publications were individually evaluated by the first author (GWR) during this procedure, and any disagreements were discussed with a third researcher (ZHF) until an agreement was reached. A color-coded risk of bias table was created, with each trial's risk for each key source of bias being classified as “low,” “medium,” or “high” (Figure 1). The results of the meta-analysis were presented together with a summary of the risk of bias that was produced by independently entering risk ratings for each trial into Review Manager (RevMan 5.4).

### 2.3 Classification criteria for MBTs subtypes

To ensure the objectivity and reproducibility of MBTs classification, this study defined clear subtype criteria based on the core intervention characteristics of each therapy (e.g., breathing pattern, targeting specificity, sound accompaniment), as detailed below:

#### 2.3.1 Yoga subtypes (classified by breathing characteristics)

Yoga interventions were subdivided according to their distinct breathing techniques, the core operational component of the practice:

Bhramari Pranayama: Referred to as “buzzing breath”; characterized by vocalized exhalation through the nose (producing a low-pitched buzzing sound similar to a bee's hum) while keeping the mouth closed, with a focus on slow, controlled airflow.

Nadi Shodhana Pranayama: Referred to as “alternate nostril breathing”; involves alternating airflow through one nostril at a time (using the thumb to block one nostril during inhalation/exhalation), with equal duration of inhalation and exhalation.

Sheetali Pranayama: Referred to as “tongue-curling breath”; involves curling the tongue into a tube shape, inhaling cool air through the curled tongue, then closing the mouth and exhaling through the nose (a cooling-focused breathing technique).

Trataka: Referred to as “gaze meditation”; differs from breath-focused yoga subtypes, focusing instead on sustained, soft gaze at a fixed target (e.g., a candle flame, a small object) to regulate attention, with no specific breathing control requirement.

Compositive Yoga Breathing (CYB): Integrates multiple basic yoga breathing techniques (e.g., combining elements of Nadi Shodhana and slow abdominal breathing) without a single dominant breathing pattern.

#### 2.3.2 Qigong subtypes (classified by intervention specificity)

Qigong interventions were subdivided based on whether they were tailored to hypertension or represented a general combination of movements:

Specific Qigong (SQ): Disease-targeted qigong practices explicitly designed or validated for hypertension management; typical examples include Baduanjin (with movements such as “shaking the head and swinging the tail to remove heart fire” that target cardiovascular regulation) and Mawangdui Daoyinshu (a traditional qigong form with gentle, hypertension-adapted movements).

Compositive Qigong (CQ): A combination of multiple qigong movements (e.g., integrating basic postures from Wuqinxi and Liuzijue) without explicit targeting of hypertension; focuses on general physical relaxation rather than disease-specific effects.

#### 2.3.3 Meditation subtypes (classified by sound accompaniment)

Meditation interventions were subdivided according to the presence or absence of sound as a guiding component:

Sound Relax Meditation (SRM): Involves guided sound cues during practice; participants repeat specific mantras (e.g., “Om”) or follow pre-recorded guided audio (e.g., soft nature sounds paired with relaxation instructions) to focus attention.

Non-sound Relax Meditation (NSRM): No external sound accompaniment; participants focus solely on internal sensations (e.g., natural breath rhythm, body scan awareness) without relying on mantras or guided sounds.

### 2.4 Evidence quality evaluation

According to the risk bias assessment tool of the systematic review provided by the Cochrane collaboration network, two reviewers evaluated the quality of the included studies. If there were any differences in the assessment results, they were decided by the third reviewer. (e assessment items included random sequence generation, concealment of distribution, blinding method, data integrity, selective reporting, and other biases. (e quality of the included studies was assessed according to three options: high risk, low risk, and unclear. If the above assessment items were low risk, the evidence grade was A; if some assessment items were low risk, the evidence grade was B; and if all assessment items were high risk, the evidence grade was C. To ensure the quality of the included studies, the studies with an evidence grade of C were excluded from this study.

The certainty of evidence for network estimates was assessed using the GRADE framework adapted for network meta-analysis, in accordance with PRISMA-NMA recommendations. Certainty was evaluated across the following domains: risk of bias, inconsistency, indirectness, imprecision, and publication bias. Risk of bias was assessed using the revised Cochrane Risk of Bias tool (RoB 2). Inconsistency was evaluated using both global and local inconsistency models. Indirectness was considered with respect to population characteristics, intervention definitions, and outcome measurements. Imprecision was judged based on confidence interval width and sample size. Publication bias was explored using funnel plot symmetry. Overall certainty of evidence for most comparisons was rated as low to moderate, primarily due to small sample sizes, sparse network connections, and wide confidence intervals.

## 3 Statistical analysis

**Figure 2:**
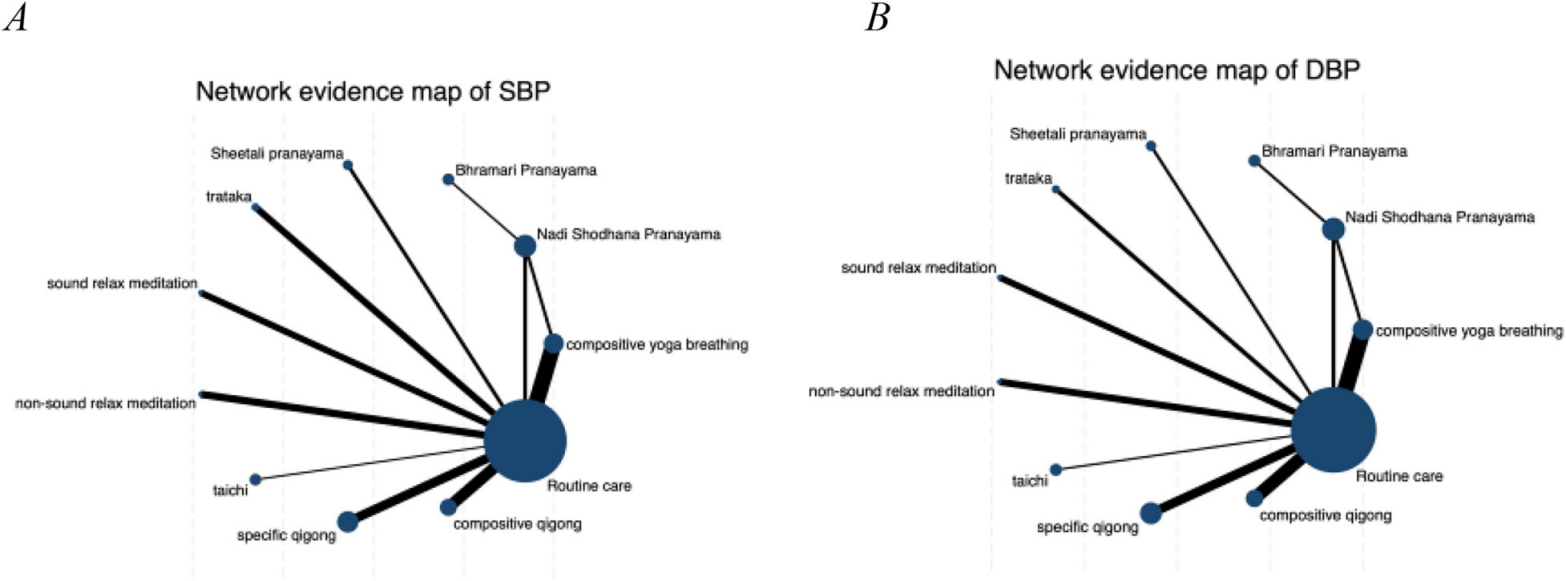
The NMA figure for SBP **(A)**, DBP **(B)**.

Network meta-analysis was performed using STATA version 18.0 under a frequentist framework, following PRISMA-NMA guidelines. The primary analysis used post-intervention SBP and DBP means and standard deviations. This decision was made because change-from-baseline data were not consistently available across all included trials. Although post-intervention analyses assume baseline comparability between randomized groups, this approach is widely accepted when baseline data are incomplete. Sensitivity analyses using change scores were conducted where sufficient data were available to assess the robustness of the findings. The different MBT interventions are represented by nodes in the network design, and the size of each node varies with the number of research participants (Figures 2A,B). Direct head-to-head comparisons are displayed by the edge lines joining the nodes. The magnitude of the effect size is correlated with the line thickness.

**Figure 3:**
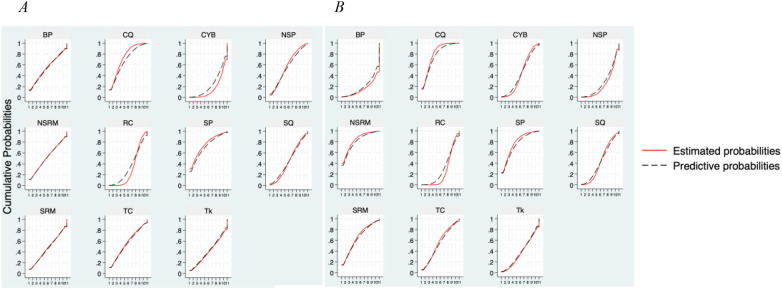
The SUCRA plot for SBP **(A)**, DBP **(B)** BP, Bhramari Pranayama; CQ, compositive qigong; CYB, compositive yoga breathing; NSP, Nadi Shodhana Pranayama; NSRM, non-sound relax meditation; RC, routine care; SP, Sheetali Pranayama; SQ, specific qigong; SRM, sound relax meditation; TC, taiChi; Tk, Trataka.

A straightforward numerical statistic that describes the cumulative ranking probability plot for every intervention as an estimated probability used to rate MBT interventions is the Surface Under the Cumulative Ranking (SUCRA) curve (Figure 3) (13). While lower SUCRA values suggest that an MBT intervention is unquestionably the worst, higher values suggest that the intervention is more likely to be at the highest level or exceptionally effective. In order to ascertain local consistency, we employed a nodal fission analysis model, fitted inconsistent and consistent models, and investigated global consistency. A consistent model was employed if there was no significant difference between direct and indirect comparisons, as shown by *p* > 0.05; if not, an inconsistent model was employed.

**Figure 4:**
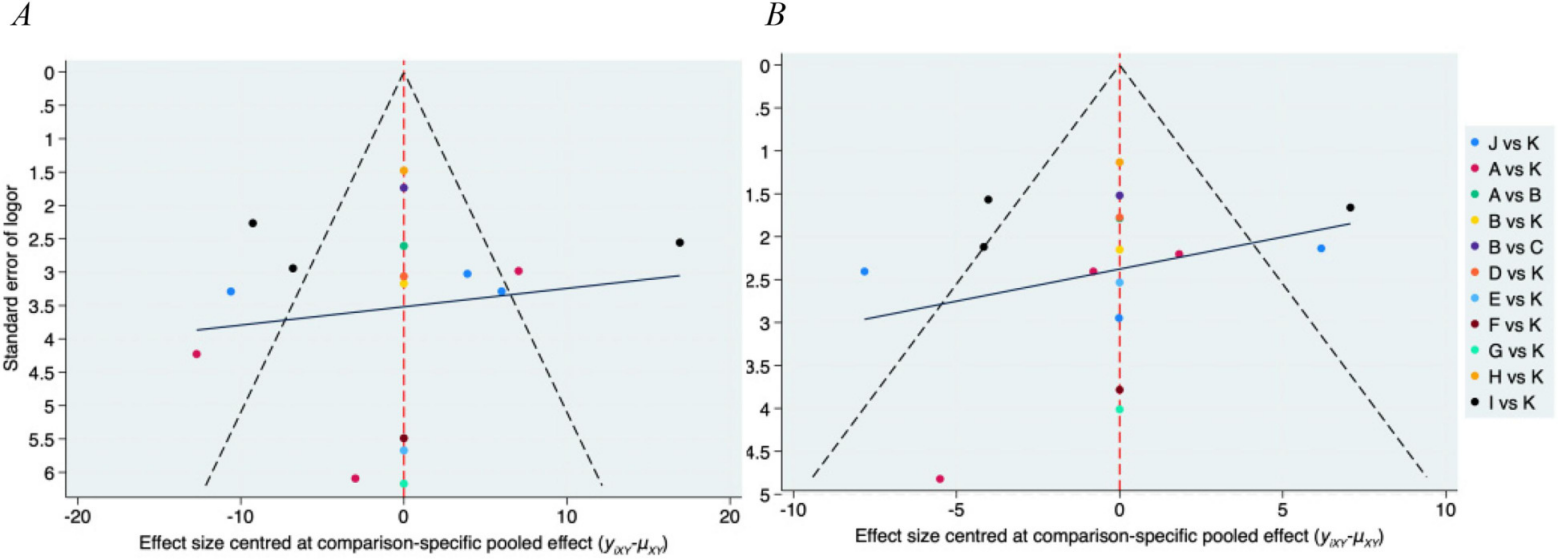
Funnel plot on publication bias of SBP **(A)**, DBP **(B).**

We created a funnel plot as a concise representation of, for instance, publishing bias, selective reporting, or other bias (Figure 4) in order to determine whether publication bias was generated (14). A straightforward scatterplot that shows the estimated intervention impact of a single trial for a specific sample size or precision is called a funnel plot. Study heterogeneity and publication bias are indicated by distribution width and symmetry, respectively. Because it is easy to see the relative disparities in effect sizes, the funnel plot has the advantage of being intuitive.

## 4 Results

### 4.1 Study identification

**Figure 5:**
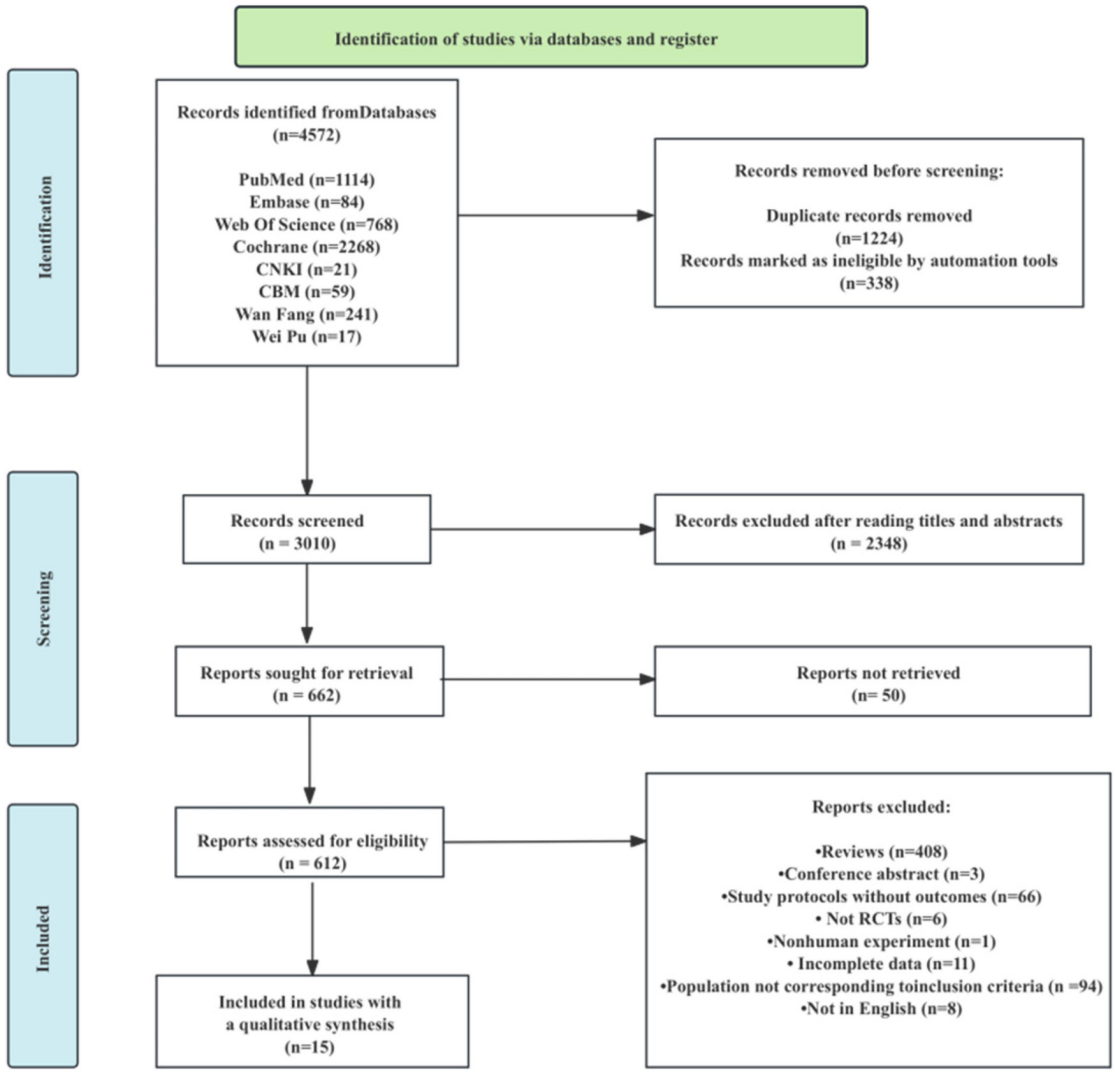
The process of selection of the eligible studies.

Using a preset search strategy, 4,572 articles in total were found. The titles and abstracts of the remaining 3010 articles were examined after duplicates and other factors were eliminated. Based on the abstract and title, 2,348 items were then eliminated from the irrelevant literature. After examining the whole text, 662 results were verified, and 647 publications were disqualified (for non-RCTs, inadequate data, conference papers, and non-compliance with the intervention, among other reasons) (Figure 5). In the end, this study comprised 15 papers (summarized in Table 1).

**Table 1:** Characteristics of included trails in this network meta-analysis.

First author, year	Magazine (name)	Sample (I/C) (Mean age)	Sample size (*n*)	lntervention	Details of interventions	Outcome indicator
Chen X, 2016 (15)	Focus on alternative & complementary therapies	48.6	46 (21\25)	I: one-minute qigong exercise C: one-minute reading session	Twice daily for 5 consecutive days	SBP; DBP
Tolbaños Roche, 2014 (16)	Complementary therapies in clinical practice	57.8	20 (10\10)	I: yoga therapy C: usual care	A total of 26*90-min sessions for three months in 26 sessions	SBP; DBP
Telles, 2013 (17)	Indian journal of physiology and pharmacology	49.7	90 (30\30\30)	I: anuloma-viloma pranayama C: read a magazine with articles of neutral content	For ten minutes	SBP; DBP
				I: breath awareness C: read a magazine with articles of neutral content		
Lin, 2021 (18)	Journal of sports medicine and physical fitness	65	99 (49\50)	I: 24-form simplified tai chi C: usual care(maintain normal dietary and exercise habits)	12 weeks of tai chi, exercise 3 times per week	SBP; DBP
Thanalakshmi, 2020 (19)	Complementary Therapies in Clinical Practice	38.5	82 (40\42)	I: practice Sheetali pranayama for a period of 3 months C: usual care	A period of 3 months(practice the pranayama for 30 min daily for 4 weeks between 7:00 to 9:00 a.m. in empty stomach)	SBP; DBP
Xiao, 2016 (20)	Journal of the American Geriatrics Society	65.6	48（24\24)	I: Qigong Ba Duan Jin C: usual care	Five times per week for 6 months	SBP; DBP
Chen, 2016 (21)	Journal of the American Geriatrics Society	66.3	60 (30\30)	I: Mawangdui Daoyinshu Qigong C: usual care	Five times per week for 6 months	SBP; DBP
Upadhyay, 2023 (22)	J Ayurveda Integr Med	31.94	100（50\50)	I: Nadi Shodhana Pranayama C: Bhramari Pranayama	For 20 min	SBP; DBP
Lee, 2003 (23)	American journal of Chinese medicine	56.45	58 (29\29)	I: Qigong(Shuxinpingxuegong) C: usual care	For 10 weeks	SBP; DBP
Pal, 2018 (24)	Asian journal of pharmaceutical and clinical research	40	50 (25\25)	I: only Yogic practices C: Nadi Shodhana Pranayama and Dhyana(meditation);Very light medications of first order primarily also prescribed which were withdrawn later (after 1 month).	For 3 months	SBP; DBP
Kusuma, 2021 (25)	Arterial hypertension (poland)	37.5	60 (30\30)	I: practice trataka for 30 min with guided instructions C: sit calmly for 30 min	For 30 min	SBP; DBP
Seer, 1980 (26)	Journal of behavioral medicine	43.2	27 (14\13)	I: SRELAX C: usual care	For 3 months;undergo training pro-cedures based on Transcendental Meditation	SBP; DBP
			27 (14\13)	I: NSRELAX C: usual care	For 3 months;undergo identical training but without a mantra	SBP; DBP
			27 (14\14)	I: SRELAX C: NSRELAX	For 3 months;undergo training pro-cedures based on Transcendental Meditation	SBP; DBP
Cheung, 2005 (27)	Journal of human hypertension	54.2	88 (47\41)	I: Goulin qigong C: conventional exercise	For 16 weeks (The subjects were asked to practise qigong for 60 min in the morning and 15 min in the evening every day for the duration of the study)	SBP; DBP
Punita, 1970 (28)	National journal of physiology, pharmacy and pharmacology	43.36	55（25\30)	I: yoga therapy C: allopathic medicines	12 weeks of yoga therapy module designed by JIPMER Institute Advanced Center for Yoga Therapy Education and Research along with the routine medical treatment	SBP; DBP
Park, 2017 (29)	Evidence-based complementary and alternative medicine	53.73	52 (25\27)	I: Dongeui qigong C: usual care	5 times per week for 12 weeks	SBP; DBP

### 4.2 study characteristics

These 15 RCTs were published between 1970 and 2023 and involved a total of 949 participants. A total of 18 intervention trials and 11 interventions were included in this NMA. Yoga was categorized based on breathing styles as, Compositive Yoga Breathing (CYB), Bhramari Pranayama (BP), Nadi Shodhana Pranayama (NSP), Sheetali pranayama (SP) and Trataka (Tk). Qigong is categorized as Specific Qigong (SQ), Compositive Qigong (CQ) based on specificity or universality. Meditation is categorized as Non-sound Relax Meditation (NSRM); Sound Relax Meditation (SRM) based on the presence or absence of sound accompaniment. In addition to this there are interventions Tai Chi (TC); Routine Care (RC). In the included studies, most of the MBTs interventions were mainly compared with conventional treatment. Among all eligible studies, 13 RCTs (15, 16, 18–25, 27–29) were two-armed experiments; 2 RCTs (17, 26) were three-arm experiments.

### 4.3 Quality assessment

Figure 1 displays the findings of the eligible RCTs' methodological quality evaluation. Despite the excellent overall quality, one experiment reported partial data, and two trials made no mention of blinding or randomized sequence generation. They were classified as “moderate risk” trials. Furthermore, four trials made reference to blinding and randomization but did not elaborate. They were classified as “low risk” trials.

### 4.4 Network meta-analysis for efficacy ranking

SUCRA values were used to estimate the probability that each intervention ranked among the most effective options for lowering SBP or DBP. These rankings reflect relative probabilities rather than definitive clinical superiority and should be interpreted in conjunction with effect sizes, confidence intervals, and the certainty of evidence.

Figures 2A,B shows the network diagrams of different MBTs to intervene in SBP and DBP in EH, and indicates that the comparisons between BP (Bhramari Pranayama), CQ (Compositive Qigong), SQ (Specific Qigong), and RC (Routine Care)—especially between SQ and RC—are common and can be considered popular interventions at present. NMA was used to compare the effects of different interventions on blood pressure in patients with EH. Figures 3A,B and Table 2A,B (cumulative rankings via SUCRA) show the relative effects of different interventions on SBP and DBP, as well as pairwise comparisons of 11 MBTs interventions for improving SBP and DBP in EH patients. On these curves (red lines), a larger area of sharp early increases indicates a higher probability of better efficacy (higher ranking), while a smaller area of slow later increases indicates lower efficacy (lower ranking).

**Table 2:** Relative effect sizes of efficacy on SBP (A), DBP (B) according to network meta-analysis.

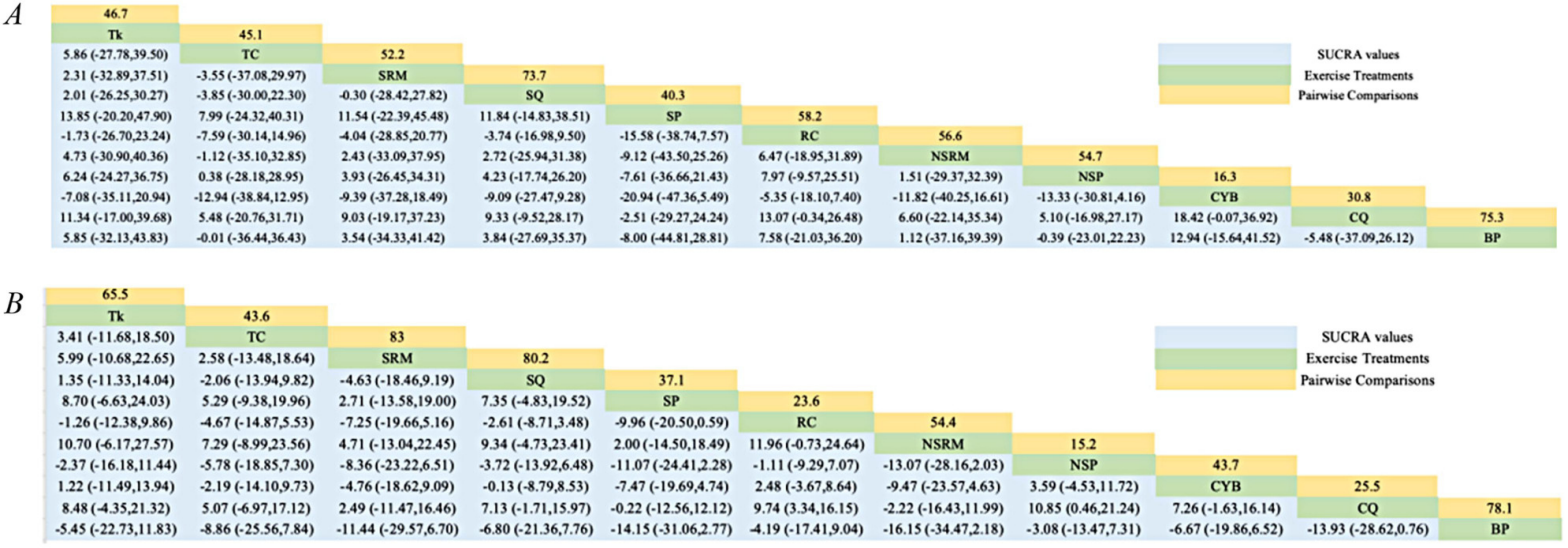

BP, Bhramari Pranayama; CQ, compositive Qigong; CYB, compositive yoga breathing; NSP, Nadi Shodhana Pranayama; NSRM, non-sound relax meditation; RC, routine care; SP, Sheetali Pranayama; SQ, specific Qigong; SRM, sound relax meditation; TC, TaiChi; Tk, Trataka.

The NMA showed that Bhramari Pranayama (BP) (SUCRA = 75.3%) and Specific Qigong (SQ) (SUCRA = 73.7%) were significantly more effective than conventional antihypertensive medication alone in lowering SBP. Specifically, BP showed a mean effect size of SMD = 12.94 (95% CI: −15.64, 41.52) for SBP reduction compared to Compositive Yoga Breathing (CYB). For DBP, the results showed that Sound Relax Meditation (SRM) (SUCRA = 83%), SQ (SUCRA = 80.2%), and BP (SUCRA = 78.1%) were significantly more effective than routine care (RC) in lowering DBP. Notably, SRM had an effect size of SMD = −7.25 (95% CI: −19.66, 5.16) for DBP reduction compared to RC. Although standardized mean differences suggest a favorable direction of effect, translating SMDs into absolute blood pressure reductions should be interpreted cautiously due to heterogeneity in baseline values and outcome variability across studies.

Moreover, Bhramari Pranayama (BP) was superior to Compositive Yoga Breathing (CYB) (SMD = 12.94, 95% CI = −15.64, 41.52) and Specific Qigong (SQ) (SMD = −8.00, 95% CI = −44.81, 28.81) in decreasing patients' SBP. Overall, Compositive Yoga Breathing (CYB) was the least effective, with the smallest reduction in SBP after intervention (SUCRA = 16.3%), so all other MBTs interventions were superior to CYB. Similarly, Sound Relax Meditation (SRM) was superior to Compositive Qigong (CQ) (SMD = 2.49, 95% CI = −11.47, 16.46) and Routine Care (RC) (SMD = −7.25, 95% CI = −19.66, 5.16) in decreasing patients' DBP. Nadi Shodhana Pranayama (NSP) was the least effective, with the smallest reduction in DBP after intervention (SUCRA = 15.2%), so all other MBTs interventions were superior to NSP.

### 4.5 Efficacy ranking

The SUCRA values and graphs show that the comparative efficacy of mind-body therapeutic interventions for SBP in patients with EH is in the following order: Bhramari Pranayama > Specific Qigong > Routine Care > Non-sound Relaxation Meditation > Nadi Shodhana Pranayama > Sound Relax Meditation > Trataka > TaiChi > Sheetali pranayama > Compositive Qigong > Compositive Yoga Breathing. The comparative efficacy of MBTs for DBP in EH patients was in the following order: Sound Relax Meditation > Specific Qigong > Bhramari Pranayama > Trataka > Non-sound Relax Meditation > Compositive Yoga Breathing > TaiChi > Sheetali pranayama > compositive Qigong > Routine Care > Nadi Shodhana Pranayama.

### 4.6 Consistency analysis

Inconsistency analysis of the global of this NMA shows *p*-values of 0.2473 and 0.8902, indicating that there is no significant inconsistency. In addition, the analysis of the node-splitting model revealed that the *p*-values were all > 0.05 indicating that there was no significant inconsistency between direct and indirect comparisons, and the consistency model was used.

### 4.7 Publication bias

Publication bias was explored using funnel plots of standardized mean differences plotted against their standard errors. Funnel plot symmetry was visually assessed to identify potential small-study effects or selective reporting.Given the limited number of studies per comparison and the sparse network structure, funnel plot interpretation remains exploratory and cannot definitively exclude publication bias (Figure 4).

## 5 Discussion

### 5.1 Key findings

The objective of this network meta-analysis (NMA) was to synthesize the available evidence from 15 randomized controlled trials (RCTs), encompassing 18 intervention arms and 11 distinct interventions, in order to compare the effects of different mind–body therapies (MBTs) on blood pressure outcomes in patients with essential hypertension (EH) through both direct and indirect comparisons. Based on SUCRA values, Bhramari Pranayama showed the highest probability of ranking among the most effective interventions for reducing systolic blood pressure (SBP) (SUCRA = 75.3%) (22), while Sound Relax Meditation demonstrated the highest probability of ranking favorably for diastolic blood pressure (DBP) reduction (SUCRA = 83%) (26). Specific Qigong ranked second for both SBP and DBP reduction (15, 21), and Bhramari Pranayama also showed a relatively high ranking for DBP reduction.

During study inclusion and intervention classification, yoga interventions were categorized according to breathing characteristics into Compositive Yoga Breathing (CYB), Bhramari Pranayama (BP), Nadi Shodhana Pranayama (NSP), Sheetali Pranayama (SP), and Trataka (Tk). Qigong interventions were classified based on specificity into Specific Qigong (SQ) and Compositive Qigong (CQ). Meditation interventions were subdivided according to the presence or absence of sound accompaniment into Non-sound Relax Meditation (NSRM) and Sound Relax Meditation (SRM). The resulting SUCRA rankings suggest that this fine-grained classification strategy provides a clearer and more nuanced understanding of the relative effects of MBT subtypes on SBP and DBP in patients with EH. This represents a novel contribution of the present NMA and distinguishes it from previous NMAs, which predominantly evaluated broad MBT categories (e.g., yoga, meditation, tai chi, and qigong) without accounting for meaningful within-category heterogeneity. While such broad classifications demonstrated overall effectiveness, they offered limited practical guidance for clinicians and patients when selecting specific interventions. By contrast, the refined categorization adopted in this NMA provides more detailed comparative evidence to support individualized selection of MBT interventions in clinical practice.

Bhramari Pranayama is a specific branch of yoga practice that has been shown to confer benefits for both physical and psychological health. The Sanskrit term Pranayama is composed of Prana (vital life force) and Yama (control), referring to yogic practices aimed at regulating the flow of vital energy and, by extension, physiological processes (30). Compared with shallow breathing, Bhramari Pranayama promotes full lung ventilation. Regular practice has been associated with favorable effects on cardiovascular and respiratory function and modulation of the autonomic nervous system, characterized by enhanced parasympathetic (vagal) activity and reduced sympathetic dominance (31). The self-generated humming sound during Bhramari Pranayama resembles mantra repetition and alters the natural respiratory rhythm. Prolonged exhalation combined with shortened inhalation may induce a state of relaxation in patients with EH, thereby contributing to reductions in both SBP and DBP.

Although meditation was initially developed for stress management, it has increasingly been applied in the management of various conditions, including depression, anxiety, and hypertension (32). Originating in ancient Vedic India, the fundamental aim of meditation is to cultivate mental and physical tranquility through inward attention and self-awareness (33). In this NMA, meditation interventions were categorized into sound-based and non-sound-based forms. Sound Relax Meditation involves sitting comfortably in a quiet environment for a designated period, during which participants gently and effortlessly repeat a specific mantra or sound. When attention is distracted by thoughts, images, bodily sensations, or emotions, participants are instructed to passively redirect attention back to the sound. The antihypertensive effects of sound-based meditation are thought to arise primarily from modulation of autonomic nervous system activity and reduction of psychophysiological stress (26). Compared with Non-sound Relax Meditation, guided sound cues may further attenuate sympathetic nervous system activation, reduce the secretion of stress-related hormones such as catecholamines, alleviate vascular tone, and improve endothelial function. Additionally, meditation has been shown to enhance parasympathetic activity and increase heart rate variability, thereby contributing to more stable blood pressure regulation.

Qigong has a history spanning several thousand years and is rooted in Taoist philosophy and traditional Chinese medicine. As a traditional mind–body therapy, it emphasizes regulation of the mind, breath, and body, with the core principle of “relaxation, tranquility, and naturalness,” and aims to improve physical and psychological function through gentle movements, breathing control, and mental focus (34). As a form of complementary and alternative medicine, qigong has been applied in the management of conditions such as hypertension and osteoporosis (23). Specific Qigong practices, characterized by targeted movements performed in a relaxed and focused state, may alleviate anxiety through psychological regulation while enhancing therapeutic effects via guided physical movements. These mechanisms are thought to suppress sympathetic nervous system activity, reduce catecholamine release, decrease vascular tone, improve microcirculation, and lower peripheral vascular resistance. Consequently, the potential benefits of Specific Qigong for EH may include blood pressure stabilization, reduced risk of complications, and improved prognosis. For example, in Liuzijue practice, the sounds “Xu,” “He,” and “Chui” are traditionally associated with regulation of the liver, heart, and kidneys, respectively, and may exert targeted effects on internal organ function (35). In Baduanjin, movements such as “shaking the head and swinging the tail to dispel heart fire” involve spinal torsion, which may help balance cardiovascular and renal function and reduce sympathetic tone (36).

### 5.2 Limitations

Several methodological limitations should be considered when interpreting these findings. Most included trials had small sample sizes, and many network comparisons were informed by single studies, resulting in imprecise estimates and low to moderate certainty of evidence under the GRADE framework. Additionally, reliance on post-intervention outcomes may introduce bias if baseline imbalances were present. Consequently, the SUCRA-based rankings should be viewed as hypothesis-generating rather than definitive guidance for clinical decision-making.

Several limitations of this NMA should be acknowledged. First, only RCTs involving patients with EH and baseline SBP ≥ 140 mmHg and/or DBP ≥ 90 mmHg were included; therefore, the findings may not be generalizable to all hypertensive populations. Second, there was substantial heterogeneity in the frequency and duration of MBT interventions across studies. Third, although an extensive literature search was conducted, inclusion was restricted to English-language publications, which may have introduced language-related selection bias. Fourth, many intervention comparisons were informed by a limited number of trials, potentially affecting the precision of the estimates.

Consequently, the accuracy and generalizability of the conclusions are limited. In addition, most included studies did not report allocation concealment, increasing the risk of selection and performance bias. The resulting rankings therefore involve inherent uncertainty and may not fully reflect real-world effectiveness. Future large-scale, rigorously designed RCTs with standardized intervention protocols are required to validate these findings.

## 6 Conclusion

To the best of our knowledge, this study is the first to thoroughly classify MBTs according to therapeutic approach and treatment efficacy. Using the NMA and SUCRA techniques, a number of direct and indirect comparisons revealed that Sound Relaxation Meditation was the most effective intervention for lowering patients' DBP and Bhramari Pranayama was the most effective intervention for lowering patients' SBP. While certain MBT subtypes demonstrated favorable relative rankings, these findings should be interpreted cautiously due to limited evidence certainty, and future large-scale, well-designed randomized trials are required to confirm these comparative effects.

## Data Availability

The raw data supporting the conclusions of this article will be made available by the authors, without undue reservation.
